# Prevalence of Human Papillomavirus (HPV) and Other Sexually Transmitted Infections (STIs) among Italian Women Referred for a Colposcopy

**DOI:** 10.3390/ijerph16245000

**Published:** 2019-12-09

**Authors:** Marianna Martinelli, Rosario Musumeci, Illari Sechi, Giovanni Sotgiu, Andrea Piana, Federica Perdoni, Federica Sina, Robert Fruscio, Fabio Landoni, Clementina E. Cocuzza

**Affiliations:** 1 Department of Medicine and Surgery, University of Milano-Bicocca, Via Cadore 48, 20900 Monza, Italy; marianna.martinelli@unimib.it (M.M.); rosario.musumeci@unimib.it (R.M.); federica.perdoni@unimib.it (F.P.); robert.fruscio@unimib.it (R.F.); dr.flandoni@gmail.com (F.L.); 2 Department of Medical, Surgical and Experimental Sciences, University of Sassari, 07100 Sassari, Italy; illarisechi@yahoo.it (I.S.); gsotgiu@uniss.it (G.S.); piana@uniss.it (A.P.); 3 ASST Monza, San Gerardo Hospital, 20900 Monza, Italy; federica.sina@gmail.com

**Keywords:** human papillomavirus, sexually transmitted infections, HPV genotypes, HPV and STI co-infections

## Abstract

Sexually transmitted infections (STIs) represent a major cause of morbidity in women and men worldwide. Human Papillomavirus (HPV) infections are among the most prevalent STIs and persistent infections with high-risk HPV (hrHPV) genotypes can cause cervical dysplasia and invasive cervical cancer. The association of other STIs with HPV cervical infection and/or dysplasia has however not yet been fully elucidated. The aim of this study was to assess the prevalence of HPV and other STIs among women presenting with an abnormal cervical cytology. Cervical infections with 28 HPV genotypes and seven other sexually transmitted pathogens were evaluated in 177 women referred for a colposcopy after an abnormal Pap smear. Positivity for at least one hrHPV genotype was shown in 87% of women; HPV 16 was the most prevalent (25.0%), followed by HPV 31 and HPV 51. The overall positivity for other STIs was 49.2%, with *Ureaplasma parvum* being the most prevalent microrganism (39.0%). Co-infections between hrHPV and other STIs were demonstrated in 17.5% of women; no significant association was demonstrated between multiple infections and the colposcopy findings. This study provides new epidemiological data on the prevalence of cervical infections associated with HPV and seven other common sexually transmitted pathogens in a population of women presenting with an abnormal cervical cytology.

## 1. Introduction

Human papillomaviruses (HPVs) are viruses known to be the primary cause of cervical cancer (CC), responsible of approximately 311,000 women’s deaths in 2018 [1]. Even though incidences of CC have decreased in recent decades, a huge burden remains, especially in low-income countries.

To date, nearly 200 different HPV types have been identified, including twelve classified by the International Agency for Research on Cancer (IARC) as oncogenic or “high-risk” HPV types (hrHPV 16, 18, 31, 33, 35, 39, 45, 51, 52, 56, 58 and 59) [2]. Although most HPV infections are benign, persistent infection with one of the carcinogenic hrHPV types is a well-established necessary cause of cervical cancer [3].

Despite HPV infections being widespread among the female population, only a small fraction of women with a hrHPV infection will progress to pre-cancer or cancer. This indicates that additional risk factors are important for carcinogenesis, potentially including viral factors such as persistent infection with specific hrHPV genotypes or the presence of concomitant pathogens able to infect the genital tract.

It is well known that HPV 16 and 18 are the two most common genotypes related to cervical cancer development [4]. In countries where HPV vaccination has been implemented with a high coverage, these HPV types have significantly decreased [5]. However, concerns have been raised that the role vacated by the vaccine-targeted types could be taken over by the non-targeted high-risk HPV types [6,7]. It is therefore important to continue monitoring HPV types′ circulation to evaluate a possible type replacement.

There is growing evidence on the role of other Sexually Transmitted Infections (STIs) as co-factors for the development of cervical cancer in HPV-positive women [8,9]. Interactions between HPV and other pathogens that share similar mucosal sites could accelerate cancer progression, enhancing HPV replication and infection persistence. For example, recent studies have reported that there is an increased risk of cervical cancer in women co-infected with HPV and *Chlamydia trachomatis* (Ct) [8,10]. Ct infection seems to determine an increased inflammatory response that can facilitate HPV’s entrance into the cervix basal membrane and favor persistent infection [11]. However, the specific role of Ct in the pathogenesis of CC is still controversial. Other microorganisms such as *Trichomonas vaginalis* (Tv), *Mycoplasma hominis* (Mh) and *Ureaplasma urealyticum* (Uu) have also been associated with cervical inflammatory processes, a situation that may facilitate the entrance of HPV [12].

The purpose of this study was to assess the prevalence and distribution of HPV genotypes among Italian women referred for a colposcopy following a recent diagnosis of abnormal cervical cytology. Moreover, the second aim was to investigate in this group of women the presence of other concomitant STIs and to determine their possible correlation with the grade of cervical lesion.

## 2. Materials and Methods

### 2.1. Study Design and Sample Collection

One hundred and seventy-seven women with a documented abnormal Pap smear were consecutively enrolled at the Gynaecology outpatient clinic of San Gerardo Hospital, Monza, Italy, between 2017 and 2019. The study protocol (Protocol: n. 305) was approved by the Ethics Committee of the University of Milano - Bicocca, Italy. A cervical sample was collected from all enrolled subjects for the molecular detection of HPV and STI pathogens. The cytological assessment was performed according to the 2001 Bethesda System [13].

The cervical samples were collected using a L-shaped FloqSwab (Copan, Brescia) and transported to the laboratory following resuspension in 20 mL of ThinPrep^®^ PreservCyt^®^ Solution (HOLOGIC™, Marlborough, MA, USA).

### 2.2. DNA Extraction, HPV and STI Detection

The DNA extraction was performed starting from 1 mL of resuspended cervical sample using NucliSENS^®^ easyMag^®^ (bioMérieux, Marcy-l’Étoile, France), an automated system for total nucleic acid extraction from clinical samples. All of the samples were processed using the “specific B” protocol, characterized by a higher final elution temperature (about 70 °C) and the use of silica beads diluted 1:2, in accordance with the manufacturer’s instructions. The nucleic acid extraction system was fully automated, except for adding the initial sample and subsequently the silica beads (125 μL), 10 min after sample lysis, reducing the potential risk of sample cross-contamination. The nucleic acid extracts were eluted in 100 μL of NucliSENS^®^ easyMag^®^ elution buffer (bioMérieux).

HPV detection and typing was performed using a commercial kit, Anyplex™II HPV28, (Seegene, Korea), which can detect 28 HPV types (i.e., 6, 11, 16, 18, 26, 31, 33, 35, 39, 40, 42–45, 51–54, 56, 58, 59, 61, 66, 68–70, 73 and 82) in 2 reaction tubes by means of real-time PCR assays on the CFX96 real-time PCR instrument (Bio-Rad, Hercules, CA, USA).

The detection of 7 STI pathogens, *Chlamydia trachomatis* (Ct), *Neisseria gonorrhoeae* (Ng), *Trichomonas vaginalis* (Tv), *Mycoplasma hominis* (Mh), *Mycoplasma genitalium* (Mg), *Ureaplasma urealyticum* (Uu), and *Ureaplasma parvum* (Up) was performed using Anyplex™ II STI-7 (Seegene, Korea).

The HPV and STI pathogen DNA detection was performed as recommended by the manufacturer using 5 μL of extracted DNA. The data recording and interpretation were done with the Seegene viewer software according to the manufacturer’s instructions.

### 2.3. Statistical Analysis

The qualitative and quantitative variables were summarized with absolute and relative (percentage) frequencies and medians (interquartile ranges, IQR), respectively. A chi-squared or Fisher exact test was performed for the qualitative variables, when appropriate. A two-tailed *p*-value less than 0.05 was considered statistically significant. The statistical software STATA version 15 (StataCorp, College Station, TX, USA) was used for all statistical computations.

## 3. Results

### 3.1. Study Population

One hundred and seventy-seven women with an abnormal cervical cytology were recruited at colposcopy. Their median (interquartile range, IQR) age was 40 (31–46) years. Patients with LSIL constituted 42.9% of the study population, followed by those with ASCUS (24.3%) and HSIL (14.1%) (Table 1). Colposcopic examinations were performed in 175 women, 74.9% (*n* = 131) of whom showed a normal colposcopy evaluation and 25.1% (*n* = 44) identified as having a high-grade cervical abnormality. Those patients with a high-grade cervical abnormality underwent treatment with conization and/or biopsy which showed the following histological findings: 11.4% (*n* = 5/44) had cervical intraepithelial neoplasia grade 1 (CIN1), 18.2% had CIN2 (*n* = 6/44), 50.0% had CIN3 (*n* = 22/44), and 6.8% had cervical cancer (*n* = 3/44), two squamous cell carcinoma and one adenocarcinoma in situ.

### 3.2. HPV Detection and Typing

**Figure 1:**
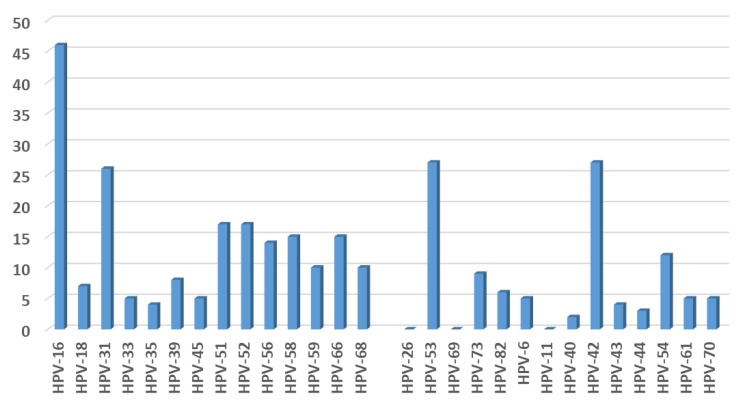
Number of HPV-type specific positive cervical samples.

In total, 132/177 (74.6%) patients were positive for at least one HPV genotype; 47% (62/132) were only positive for high-risk HPV (hrHPV) types, 40.2% (53/132) for co-infection with hrHPV and low-risk HPV (lrHPV) types, and 12.9% (17/132) for only lrHPV types (Figure 1). HPV 16 was the most prevalent hrHPV, followed by HPV 31, HPV 51 and HPV 52. HPV 53 and HPV 42 were the most frequent lrHPV types.

**Figure 2:**
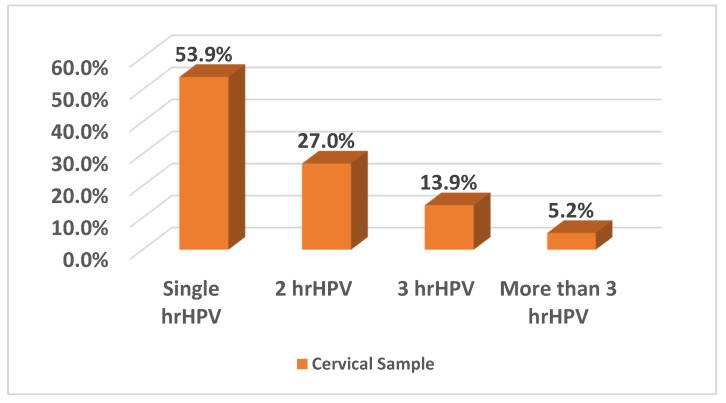
Mono- and multiple infections caused by hrHPV types.

In total, 53.9% (62/115) of the women were infected with only one hrHPV type, whereas multiple hrHPV infections were detected in 46.1% (53/115) of patients (Figure 2).

A total of 68.2% (15/22) of those infected with only HPV 16 had a high-grade cervical abnormality (Table 2); 91.7% (55/60) of the women found to be hrHPV negative had a negative colposcopy.

### 3.3. STI Prevalence

**Figure 3:**
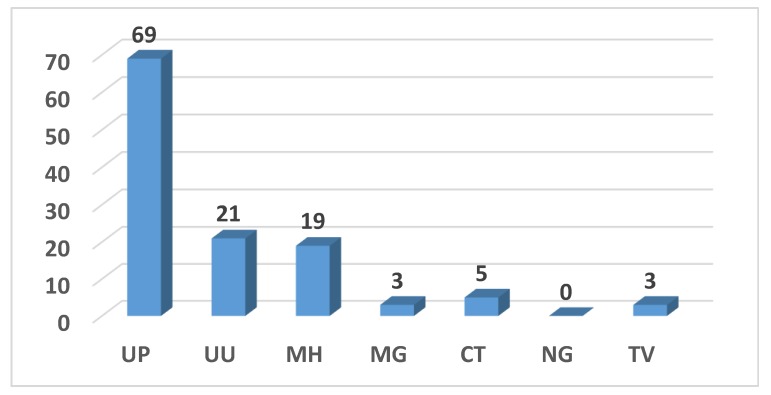
Prevalence of STI pathogens.

Overall, the positivity for at least one STI was 49.2% (87/177). *Ureaplasma parvum* was the most prevalent pathogen (69/177, 39.0%), followed by *Ureaplasma urealyticum* (21/177, 11.9%) and *Mycoplasma hominis* (19/177, 10.7%). *Chlamydia trachomatis* was identified in 2.8% (5/177) of women, whereas *Mycoplasma genitalium* and *Trichomonas vaginalis* were found in three (1.7%) of the patients (Figure 3).

**Figure 4:**
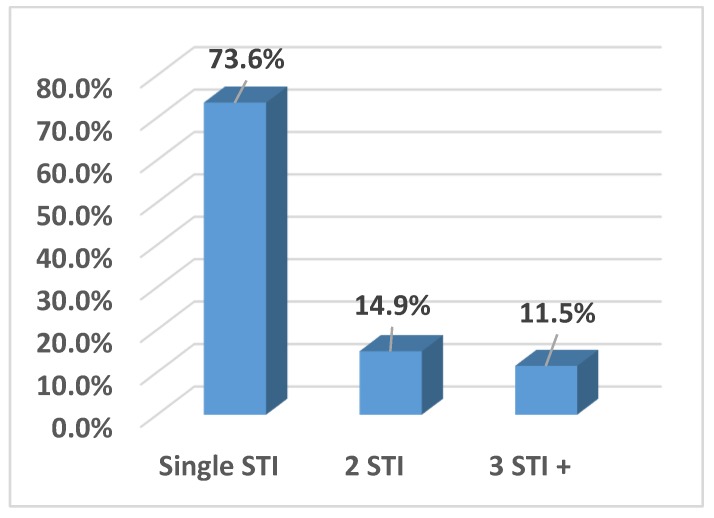
STIs multiple infections.

The majority of the women with a STI (*n* = 87) were infected by a single pathogen (69, 73.6%), whereas 14.9% (13/87) and 11.5% (10/87) showed two or three or more STIs, respectively (Figure 4). A total of 65 (36.7%) women were HPV/STI co-infected. *Ureaplasma parvum* (37/65, 56.9%) was the most prevalent pathogen in co-infected patients, followed by *Chlamydia trachomatis*, *Mycoplasma genitalium*, and *Trichomonas vaginalis*.

Among the women with a positive colposcopy result, 54.5% (24/44) had STIs (Table 3). In those women found to be positive for both hrHPV infection and colposcopy findings, 30.8% (12/39) were co-infected with *Ureaplasma parvum*, 10.3% (4/39) with *Ureaplasma urealyticum*, and 15.4% had multiple STIs (6/39).

## 4. Discussion

HPV infection is the necessary but not sufficient cause for cervical cancer. Other cofactors can play a role, influencing HPV persistence and cervical disease progression.

The overall prevalence of both high-risk and low-risk HPVs in our study population of women referred for colposcopy after a recent abnormal Pap test was found to be 74.6%, with 65% (115/177) of them being positive for at least one hrHPV type. The high HPV positivity rates found in this selected population is in keeping with the range of HPV positivity previously reported among the patients with the same characteristics [14,15,16,17]. The prevalence rates are also influenced by the pre-analytical procedure for sample processing and the HPV test used for viral DNA detection [14,15,16,17]. For example, a Norwegian study reported an HPV DNA positivity of 82.7% using the same Anyplex™II HPV28 test [14]. Tewari and colleagues found a hrHPV prevalence of 58% using Cobas 4800 HPV test and 53% using Aptima HPV assay, respectively [17]. In two other studies conducted in our laboratory, using “in-house” real time assays able to detect only seven out of the 12 hrHPV genotypes, we observed an HPV prevalence of 44.2% and 34.3%, respectively [15,16].

HPV 16, 31, 51 and 52 were found to be the most prevalent hrHPVs detected. Baasland et al. [14] reported similar results, with the exception of HPV 51, which appears to be more frequent in Italy. Previously published studies have in fact reported a high prevalence of the HPV 51 genotype in Italian women [15,18,19]. The high prevalence of HPV 16 is in keeping with the worldwide spread of this genotype, not only among women but also in the male population [6,9,14,20,21].

The majority of the women with a HPV 16 single infection were found to have a high-grade cervical abnormality at colposcopy examination (68.2%), demonstrating again the principal role of this genotype in precancerous lesion progression.

Overall, positivity for one or more of the seven STI pathogens under study was demonstrated in 49.2% of women, with *Ureaplasma parvum* found to be the most frequently identified pathogen (69/177, 39.0%), followed by *Ureaplasma urealyticum* (21/177, 11.9%). 

*Chlamydia trachomatis* was identified in 2.8% of women and was always in co-infection with other STI pathogens. This positivity rate was low compared to the 12.8% of *Ct* prevalence that our group found in a previous study conducted on women with a recent history of cervical dysplasia [15] and other data reported in the literature regarding the diffusion of this pathogen among sexually active women (ranging from 1.8% to 10.4%) [22,23,24,25,26,27].

Several previously published studies reported a very high percentage of *Ureaplasma spp.* infection in female genital tracts. A study conducted in Japan showed an *Ureaplasma parvum* prevalence of 41.7% in women attending their first prenatal visit [23]; another study, carried out in Slovenia, showed that significantly more women aged 25 years and younger were infected with *U. urealyticum* (23.4%) compared to those aged above 25 years (9.2%), irrespective of symptoms [28]. Other studies conducted in Italy have reported positivity rates for *Ureaplasma parvum* of almost 25% in vaginal specimens [22] and 38.3% in cervical samples [29].

As a result of the high prevalence of *Ureaplasma* spp. and *Mycoplasma* spp. detection in the genital tracts of sexually active individuals and their unclear clinical role in lower urogenital tract infections, a recent position statement from the European STI Guidelines Editorial Board has indicated that the routine testing and treatment of asymptomatic or symptomatic men and women for *M. hominis*, *U. urealyticum* and *U. parvum* are not recommended [30].

However, several recent studies investigating the role of cervical co-infections of *Ureaplasma* spp. and other STIs in association with hrHPV, have supposed that these could be possible cofactors interacting with HPV in the development of precancerous and cancerous cervical lesions [31,32,33,34,35,36]. 

Moreover, a recent systematic review and meta-analysis aimed at investigating the association between female genital mycoplasmas and HPV infection, abnormal cervical cytopathology, and cervical cancer, concluded that *Ureaplasma urealyticum* and *Ureaplasma parvum* were associated with a significantly increased risk of overall HPV infection (OR 1.57; OR 3.02, respectively). Furthermore, infections with *U. urealyticum*, *U. parvum*, and *Mycoplasma hominis* were also found to be associated with a significantly increased risk of abnormal cervical cytopathology (OR 1.51; OR 1.41; OR 1.48, respectively) [37].

In the present study, infections with multiple hrHPV types were detected in 46.1% (53/115) of the HPV-positive patients; a total of 65 (36.7%) women were also found to be co-infected with HPV and STIs. In these co-infections, *Ureaplasma parvum* (37/65, 56.9%) was the most prevalent sexually transmitted pathogen, followed by *Chlamydia trachomatis*, *Mycoplasma genitalium*, and *Trichomonas vaginalis*. In spite of the relatively high prevalence of hrHPV and STIs co-infections observed in the population studied, no statistically significant association was found between co-infections and positive colposcopy findings in these women.

Only in depth in vitro studies may confirm whether *Ureaplasma* spp. and/or other STIs are real cofactors or are just “followers”, taking advantage of the immune tolerance and abnormal regulation of the cell cycle control generated by HPV. Moreover, future longitudinal clinical studies in women with an abnormal cervical cytology will allow us to better evaluate the relationship between lower genital tract infections, co-infections, and high-risk human papillomavirus infections.

## 5. Conclusions

The results of this study have demonstrated, as expected, a high prevalence of hrHPV infections in women referred for colposcopy, but also high rates of co-infections with *Ureaplasma parvum* and other STIs. Although a statistically significant association between co-infections between hrHPV and other STIs with positive colposcopy findings could not be demonstrated in the studied population, the high prevalence of STIs found in hrHPV-positive women, who are at higher risk of developing cervical disease, indicate that screening for genital infections in this population may be important. This would allow not only to prevent other STDs and their sequelae, but also reduce any potential influence of concomitant microorganisms on HPV infection. Future longitudinal studies investigating the role of STIs as cofactors in HPV mediated cervical carcinogenesis are warranted.

## Figures and Tables

**Table 1:** Pap test smear results.

Cytology	*n*	%
HSIL	25	14.1%
ASCH	14	7.9%
LSIL	76	42.9%
AGCUS	9	5.1%
ASCUS	43	24.3%
NILM	10	5.6%
Total	177	

HSIL (High grade squamous intraepithelial lesion); ASCH (Atypical squamous cells—cannot exclude HSIL); LSIL (Low-grade squamous intraepithelial lesion); AGCUS (Atypical Glandular Cells of undetermined significance); ASCUS (Atypical squamous cells of undetermined significance) NILM (Negative for Intraepithelial Lesion or Malignancy).

**Table 2:** Differences between patients with positive and negative colposcopy results based on hrHPV infection.

Women with Available Colposcopy Result (n = 175)	Positive Colposcopy n (%)	Negative Colposcopy n (%)	*p*-Value *
Single HPV 16 (n = 22)	15 (34.0%)	7 (5.3%)	<0.0001
Single HPV 18 (n = 1)	1 (2.3%)	0 (0.0%)	0.31
HPV 16 + other hrHPV (n = 24)	5 (11.4%)	19 (14.5%)	0.59
HPV 18 + other hrHPV (n = 6)	2 (4.6%)	4 (3.1%)	0.67
Single other hrHPV (n = 39)	10 (22.7%)	29 (22.1%)	0.93
Multiple other hrHPV (n = 23)	6 (13.6%)	17 (13.0%)	0.92
hrHPV Negative (n = 60)	5 (11.4%)	55 (42.0%)	<0.0001
	44 (25.1%)	131 (74.9%)	

* Chi-squared or Fisher exact test, when appropriate.

**Table 3:** Correlation between STI detection and colposcopy findings and hrHPV positivity.

Women with Available Colposcopy Result (n = 175)	Positive Colposcopy n. (%)	Negative Colposcopy n. (%)	*p*-Valhue *
Single STI (n = 64)	18 (40.1%)	46 (35.1%)	0.56
Multiple STI (n = 23)	6 (13.6%)	17 (13.0%)	0.92
Negative (n = 88)	20 (45.5%)	68 (51.9%)	0.46
**hrHPV positive women infected also with (n = 114):**			
Up (n = 37)	12 (30.8%)	25 (33.3%)	0.79
Uu (n = 6)	4 (10.3%)	2 (2.7%)	0.09
Mh (n = 2)	0 (0.0%)	2 (2.7%)	0.30
Mg (n = 0)	0 (0.0%)	0 (0.0%)	-
Ct (n = 0)	0 (0.0%)	0 (0.0%)	-
Ng (n = 0)	0 (0.0%)	0 (0.0%)	-
Tv (n = 0)	0 (0.0%)	0 (0.0%)	-
Multiple STIs (n = 20)	6 (15.4%)	14 (18.7%)	0.66
Negative (n = 49)	17 (43.6%)	32 (42.7%)	0.93

* Chi-squared or Fisher exact test, when appropriate.

## Abbreviations

STDs	sexually transmitted disease
HPV	Human Papillomavirus
HSIL	High grade squamous intraepithelial lesion
ASCH	Atypical squamous cells-cannot exclude HSIL
LSIL	Low-grade squamous intraepithelial lesion
AGCUS	Atypical Glandular Cells of undetermined significance
ASCUS	Atypical squamous cells of undetermined significance
NILM	Negative for Intraepithelial Lesion or Malignancy
CIN1	cervical intraepithelial neoplasia grade 1
CIN2	cervical intraepithelial neoplasia grade 2
CIN3	cervical intraepithelial neoplasia grade 3
Ng	*Neisseria gonorrhoeae*
Tv	*Trichomonas vaginalis*
Mh	*Mycoplasma hominis*
Mg	*Mycoplasma genitalium*
Uu	*Ureaplasma urealyticum*
Up	*Ureaplasma parvum*
Ct	*Chlamydia trachomatis*
PCR	polymerase chain reaction
